# Mutation choice biases the structure of empirical fitness landscapes

**DOI:** 10.64898/2026.09.28.754994

**Published:** 2026-10-22

**Authors:** Caelan Brooks, Michael M. Desai, Maryn O. Carlson

## Abstract

Evolution can be thought of as taking place in a fitness landscape which maps each genotype to a fitness value. This abstraction has motivated efforts to quantify the statistics of random fitness landscapes with different amounts and types of interactions among loci, or epistasis. High-throughput experiments measuring the phenotypes of thousands of genotypes promise to bridge this theory with data. Yet thousands is still small relative to genotype space. Moreover, experiments often assay a highly non-random subset of genotypes. For example, a common approach is to assay all 2 ^*M*^ intermediates between ancestral and evolved sequences differing by *M* ≪ *L* mutations. Here, we ask how well the statistics of a landscape constructed under distinct “ascertainment” protocols recapitulate those of a random subset of the global landscape in several random landscape models. We show that, in most landscapes, selecting mutations that occurred along an adaptive trajectory inflates the variance explained by lower-order terms, reduces the number of local maxima, and increases the number of accessible paths relative to a random fitness landscape. Selecting the most beneficial or deleterious mutations instead distorts these statistics in ways that depend more critically on the amount of epistasis in the global landscape. We then use three large-scale mutagenesis datasets to “simulate” ascertainment in more realistic settings. In two of these, differences among schemes are likely explained by measurement noise and global nonlinearities; in the third, these differences at least partly reflect biased sampling of genuine interactions among loci.

